# Spontaneous Perforation of Meckel's Diverticulum in a Young Adult Male: A Case Report and Review of the Literature

**DOI:** 10.7759/cureus.53598

**Published:** 2024-02-05

**Authors:** Mahmoud S Aly, Zohaib Jamal

**Affiliations:** 1 Department of Surgery, Wrightington, Wigan and Leigh NHS Foundation Trust, Wigan, GBR

**Keywords:** acute abdomen, congenital anomaly, perforation, appendicitis, meckel´s diverticulum

## Abstract

Meckel's diverticulum, a congenital defect that affects about 2% of the population, is a remnant of the embryologic vitelline duct. Perforated Meckel's diverticulum, a rare consequence of an already rare disease process, frequently presents and is diagnosed as a perforated appendix. We report a case of a 28-year-old male who presented with a two-day history of right-sided lower abdominal pain associated with nausea. The abdominal examination revealed a soft, nondistended abdomen with tenderness in the right iliac fossa. A CT scan of the abdomen showed a normal appendix and inflammation of Meckel's diverticulum without any signs of perforation. Bowel exploration through a small midline incision indicated the presence of a highly inflamed Meckel's diverticulum with localized perforation 75 cm from the ileocecal valve. A resection of 15 cm of the small bowel and an end-to-end primary anastomosis were performed. The patient had an uncomplicated recovery and was discharged after a five-day admission to a surgical ward. This case report illustrates the significance of keeping Meckel's diverticulum as a differential diagnosis in all the patients who present with an acute abdomen.

## Introduction

The name *Meckel's diverticulum *derives its origin from Johann Friedrich Meckel, a German comparative anatomist, who was the first person to provide a description of its embryology and pathological characteristics in 1809. He reported Meckel's diverticulum to be an anomaly of the gastrointestinal tract formed as a result of the failure of complete obliteration of the vitelline duct in the fifth gestational week [1]. In the literature, it is commonly referred to as the rule of 2's: present in 2% of the population, in 2 feet proximity of the ileocecal valve, 2 inches in total length, containing two kinds of heterotopic mucosa, and mostly presenting before the two years of age [2,3]. It is the commonest of congenital anomalies of the gastrointestinal tract; however, it is usually found incidentally at the time of laparotomy [3,4].

Patients with Meckel's diverticulum usually present at a young age. It very rarely manifests itself symptomatically and has an estimated 4% lifetime complication rate. However, when it does, it typically presents as gastrointestinal bleeding in children, and in adults, obstruction caused by intussusception or adhesive bands is the most common presentation, accounting for approximately 22%-50% of complicated Meckel's diverticulum presentations [5]. Some of the other common complications associated with Meckel's diverticulum include bleeding, intussusception, inflammation, and neoplasms. Perforation of Meckel's diverticulum is a very rare presentation in both children and adults, accounting for only 0.5% of total presentations, and is usually seen in older women [6, 7]. The Meckel's diverticulum may perforate spontaneously or as a result of pressure necrosis of the diverticulum wall and foreign body irritation. [8] The signs and symptoms of perforated Meckel's diverticulum, which include fever, nausea, vomiting, right iliac fossa pain, and peritoneal irritation, usually mimic those of perforated acute appendicitis. Asymptomatic Meckel's diverticulum has a similar incidence in males and females. However, studies have shown an incidence ratio of 1-4:1 male-to-female in symptomatic presentations [9].

## Case presentation

This is the case of a 28-year-old male, typically fit and well. He presented to the emergency department with a two-day history of right-sided lower abdominal pain resistant to analgesia and associated with nausea but no vomiting. The patient did not have any change in his normal bowel habits and did not have any urinary symptoms.

On physical examination, the abdomen was soft, with minimal tenderness in the right iliac fossa. No distention or rebound tenderness was noted. He remained vitally stable. Differential diagnoses of acute appendicitis, urinary tract infection, renal stones, and mesenteric lymphadenitis were established based on the history and examination findings.

**Figure 1:**
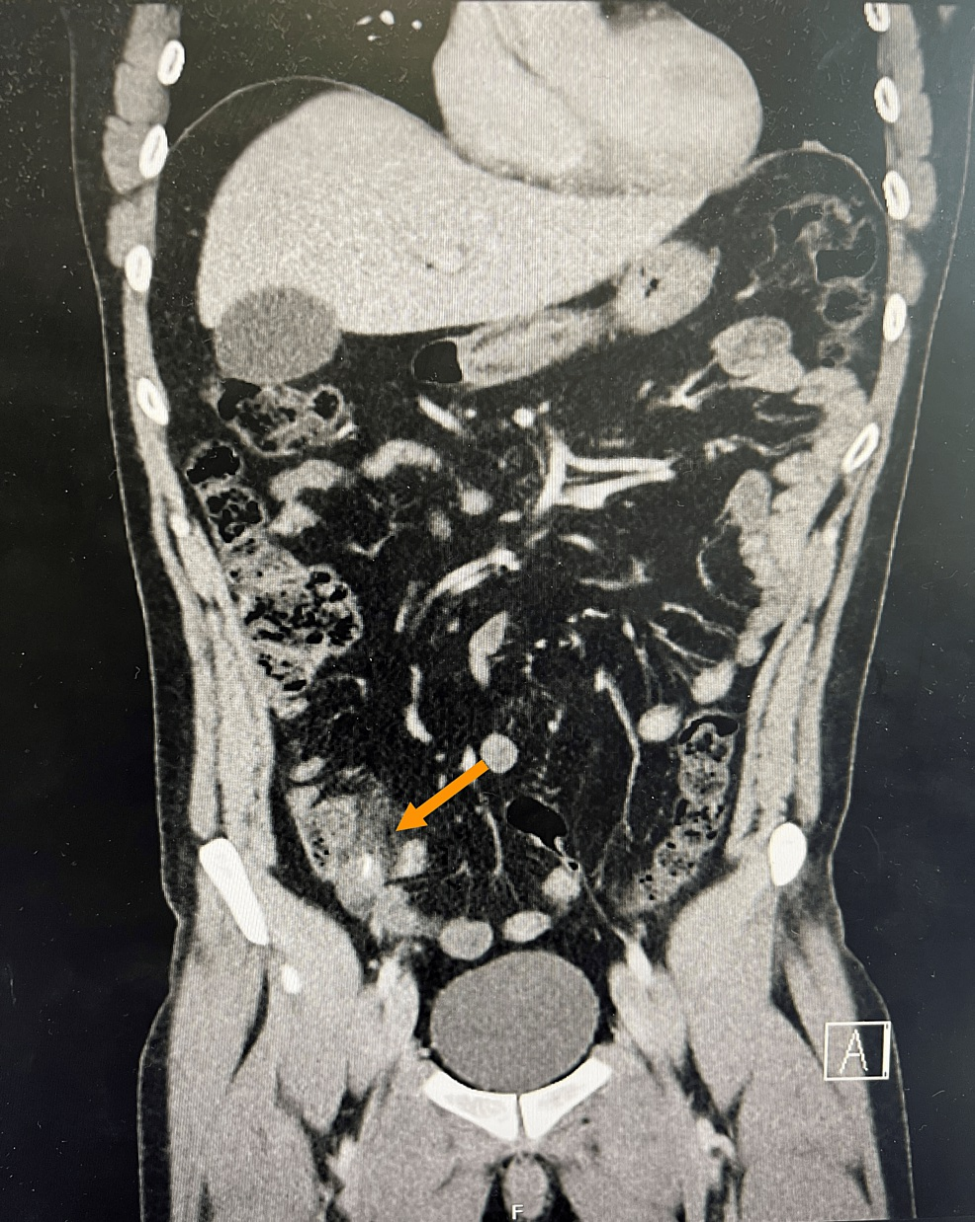
A coronal cut section of the CT abdomen and pelvis reveals an inflammatory condition and central calcification within a hollow viscus.

**Figure 2:**
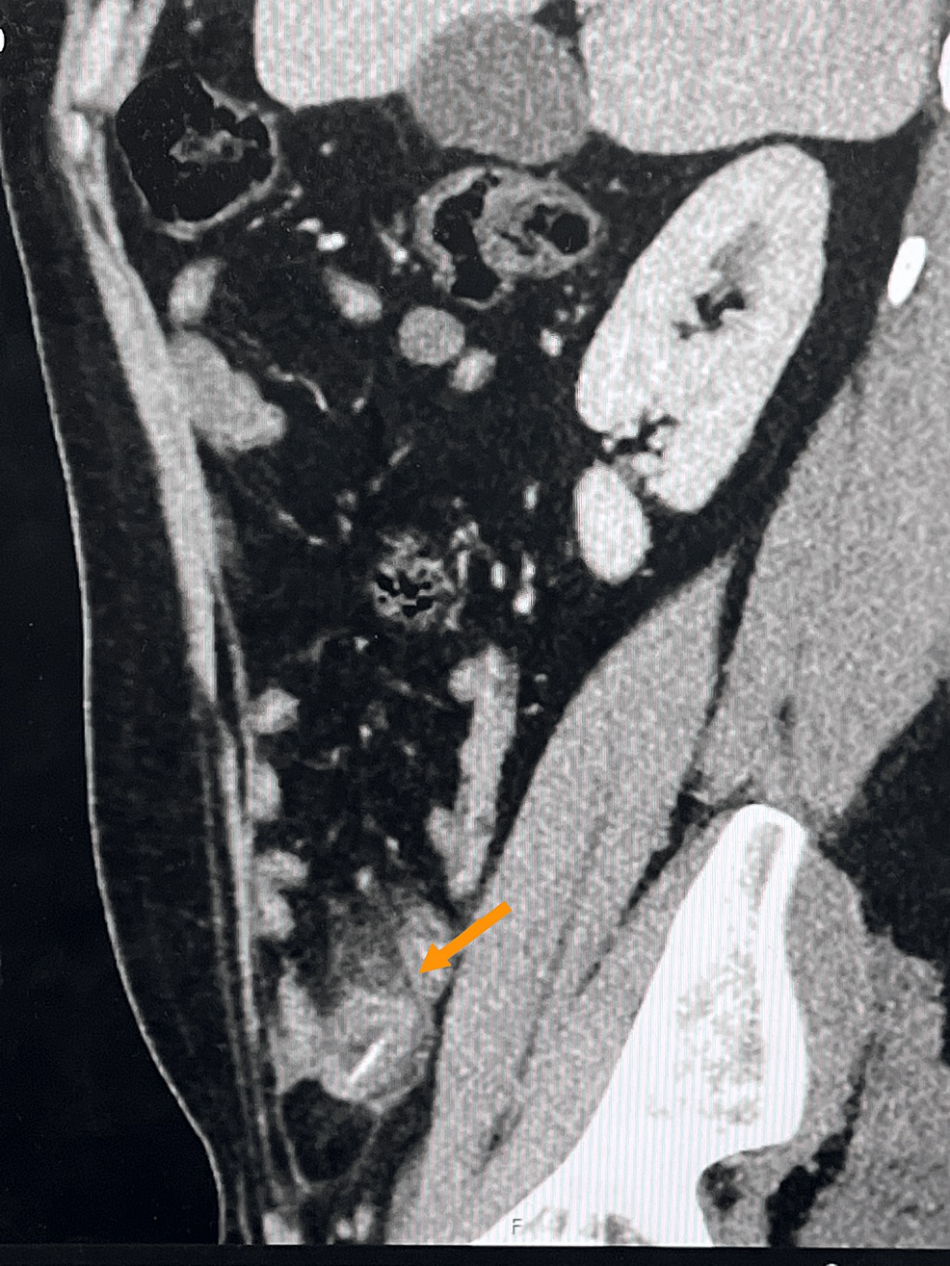
A sagittal cut section of the CT abdomen and pelvis demonstrates a perforated Meckel's diverticulum.

Initial blood tests showed a hemoglobin level of 14.1 g/dL, a white blood cell count (WBC) of 7.1 x 10^9 ^L^-1^, a C-reactive protein (CRP) of 20, and normal renal function. A computed tomography (CT) scan with intravenous (IV) contrast was performed based on clinical examination, which was reported as inflammation related to a hollow viscus with calcification, the abnormal pathology being distant from the cecal pole, and a likely normal appendix. The abnormality is probably related to the distal ileum and possibly represents an inflamed Meckel's diverticulum with no perforation, free fluid, or fluid collection. The CT images are shown in Figures 1-2.

A definitive diagnosis of complicated Meckel's diverticulum was established, and surgical resection was offered to the patient with a possibility of stoma formation. Formal exploration of the bowel through a small midline incision revealed the presence of highly inflamed Meckel's diverticulum with localized perforation, 75 cm from the ileocecal valve. The appendix was identified to be normal. A resection of 15 cm of the small bowel, including the pathology, was performed and sent for histopathology. The end-to-end primary anastomosis was conducted using linear staplers, and an intra-abdominal drain was left. The patient’s recovery was uneventful overall; he was admitted to a surgical ward and was kept nil by mouth on IV fluids for postoperative day 1. Follow-up blood investigations showed a CRP of 126 with normal WBC. He was started on clear fluids on postoperative day 2.

On postoperative day 3, he started to develop headaches and diplopia, which were more likely due to patient-controlled analgesia. However, a CT head was performed and showed normal findings. On postoperative day 4, the patient passed motion and was started on a normal diet. His wound was clean with no signs of active infection. His drain output showed only minimal serosanguinous fluid, which decreased significantly in amount and became just traces on postoperative day 5. On postoperative day 5, blood tests were repeated and were completely normal. He was discharged on analgesia and oral antibiotics with a plan to be reviewed in the surgical ambulatory care unit (SACU). The patient presented to the SACU and was eating and drinking, opening his bowels normally, and did not have any complaints of abdominal pain. His abdominal examination was unremarkable, and the wound was healthy. He was discharged home with skin clips to be removed 12-14 days from the date of the operation in the community and advised to have a patient-initiated follow-up if any complications arise.

A histopathology report of the resected specimen showed an area of punched-out type transmural ulceration associated with marked acute inflammation with abscess formation which extends almost entirely through the peri-intestinal fat with neutrophils lying very close to the serosal surface. There was no evidence of granuloma formation, dysplasia, or neoplasia. The appearance is in keeping with the clinical impression of inflamed Meckel's diverticulum.

**Figure 3:**
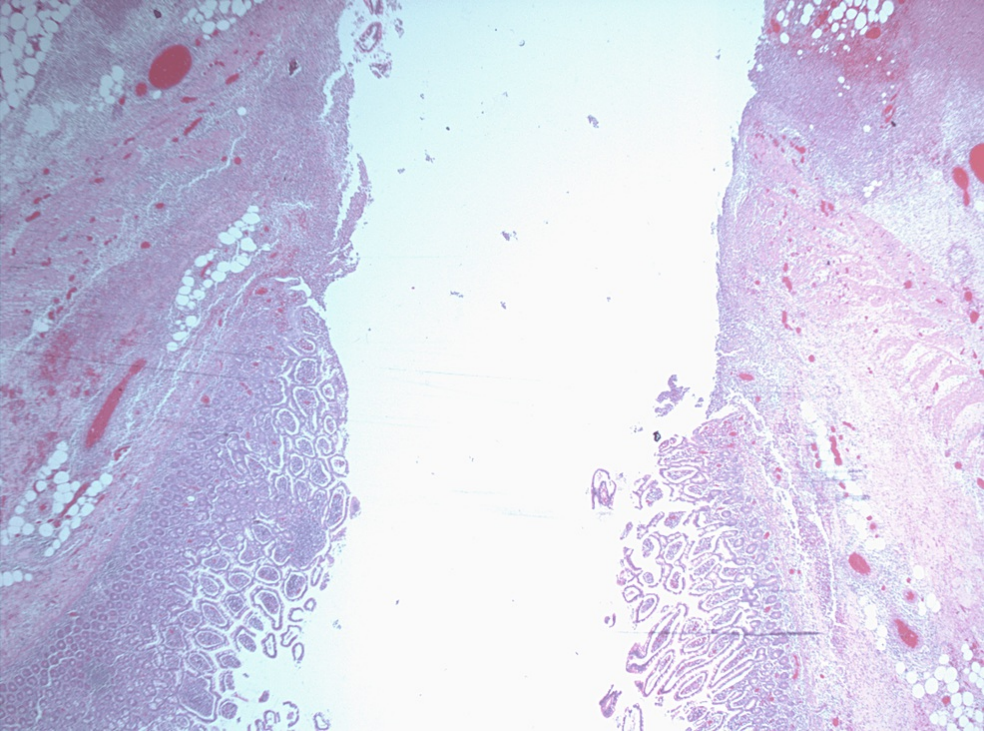
Histopathology image of the specimen.

## Discussion

We present an interesting case of a 28-year-old male presenting with right-sided lower abdominal pain and diagnosed with a perforated Meckel's diverticulum, which is particularly noteworthy due to its unusual occurrence in this age group and gender and the challenge it poses in differential diagnosis. The patient initially presented with symptoms suggestive of acute appendicitis, and while CT findings indicated inflammation of Meckel's diverticulum without perforation, intraoperative exploration revealed a perforated Meckel's diverticulum, highlighting the diagnostic challenge and the need for surgical intervention. Acute abdomen is an emergency that requires immediate surgical assessment. Numerous factors, such as intestinal blockage, vascular occlusion, inflammation, or infection, can be the cause. One of the many differential diagnoses that need to be taken into account in this clinical presentation is a complicated Meckel's diverticulum. Meckel's diverticulum is formed when the small bowel undergoes dysgenesis while developing in connection to the embryological yolk sack. It forms due to incomplete obliteration of the vitelline duct that occurs during the fifth and eighth weeks of pregnancy. 

Perforation of Meckel's diverticulum is a rare presentation and has an incidence of 0.5% of total presentations, especially in older females [6,7,10]. However, Chen et al. reported a surprisingly high 7.3% incidence of Meckel's perforation in children [11]. A study by Park et al. conducted at the Mayo Clinic reported a 10%-12% perforation rate in symptomatic patients [12]. Very rarely, incidences of perforation secondary to blunt trauma to the abdomen have been recorded. Park and Lucas reported the first case in 1970 [13].

Due to the similar clinical and imaging characteristics of other acute abdominal surgical conditions, a preoperative diagnosis of complicated Meckel's diverticulum may be challenging. Symptomatic Meckel's diverticulum has only been accurately diagnosed between 5.7% and 13% of the time and reported approximately 55 times less than acute appendicitis [14]. Although the majority of Meckel's diverticula are discovered intraoperatively, it is interesting to note that Ueberrueck et al. reported that 19.1% of their 9,793 appendectomies had no search for Meckel's diverticulum, suggesting the possibility of it being overlooked in the operating theaters despite being an important differential of acute appendicitis [15]. Adults with perforated MD were the subject of a study by Ding et al., and it revealed that out of the 60% of patients diagnosed with perforated appendicitis, 13% had perforated Meckel's diverticulum [16].

A complicated Meckel's diverticulum, just like in our patient, almost always requires a surgical intervention. Farah et al. also reported a case of a 26-year-old male who was found to have a spontaneous perforation of Meckel's diverticulum and underwent emergency laparotomy [17]. Another such case of spontaneous perforation of Meckel's diverticulum in a 49-year-old male has been described by Jain in which bowel resection followed by an end-to-end anastomosis was performed as a definitive management [18]. A very interesting case in a 54-year-old female of similar pathology has been reported by Fraser et al. where the patient initially had an appendectomy at a different hospital and later presented with signs and symptoms suggestive of stump leak. Exploratory laparotomy performed in this patient also revealed an inflamed Meckel's diverticulum, which was managed with bowel resection and anastomosis as well [9]. Liu and Wu also reported a similar case of spontaneously perforated Meckel's diverticulum in a 20-year-old male patient managed surgically [19].

## Conclusions

In summary, because of the presenting similarity and difficult preoperative diagnosis, perforated Meckel's diverticulum should be considered as a differential diagnosis for individuals who experience sudden onset abdominal pain that mimics acute appendicitis. After the diagnosis is established, then the management of spontaneously perforated Meckel's diverticulum involves resection of the bowel involved followed by end-to-end anastomosis. Decisions regarding the surgical approach for treatment should consider the surgeon's expertise and preference, the patient's individual characteristics, the results of the clinical examination, and the hemodynamic status.
